# Osteocalcin and Its Potential Functions for Preventing Fatty Liver Hemorrhagic Syndrome in Poultry

**DOI:** 10.3390/ani13081380

**Published:** 2023-04-18

**Authors:** Wenjun Tu, Yuhan Zhang, Kunyu Jiang, Sha Jiang

**Affiliations:** 1 Joint International Research Laboratory of Animal Health and Animal Food Safety, College of Veterinary Medicine, Southwest University, Chongqing 400715, China; 2 Immunology Research Center, Medical Research Institute, Southwest University, Chongqing 402460, China

**Keywords:** chicken, osteocalcin, Fatty Liver Hemorrhagic Syndrome

## Abstract

**Simple Summary:**

Fatty liver hemorrhage syndrome is one of the main metabolic diseases in laying hens, which leads to lipid accumulation, fragile liver, rupture bleeding, and sudden death. Osteocalcin, a kind of noncollagenous protein, has recently been found to protect chickens from the disease. However, the most studies have focused on the use of osteocalcin in mammals. In this review, we try to outline the functions of osteocalcin in fatty liver hemorrhage syndrome in poultry based on the recent outcomes.

**Abstract:**

Osteocalcin (OCN) is synthesized and secreted by differentiating osteoblasts. In addition to its role in bone, OCN acts as a hormone in the pancreas, liver, muscle, fat, and other organs to regulate multiple pathophysiological processes including glucose homeostasis and adipic acid metabolism. Fat metabolic disorder, such as excessive fat buildup, is related to non-alcoholic fatty liver disease (NAFLD) in humans. Similarly, fatty liver hemorrhage syndrome (FLHS) is a metabolic disease in laying hens, resulting from lipid accumulation in hepatocytes. FLHS affects hen health with significant impact on poultry egg production. Many studies have proposed that OCN has protective function in mammalian NAFLD, but its function in chicken FLHS and related mechanism have not been completely clarified. Recently, we have revealed that OCN prevents laying hens from FLHS through regulating the JNK pathway, and some pathways related to the disease progression have been identified through both in vivo and vitro investigations. In this view, we discussed the current findings for predicting the strategy for using OCN to prevent or reduce FLHS impact on poultry production.

## 1. Fatty Liver Hemorrhage Syndrome

Fatty liver hemorrhage syndrome (FLHS) of laying hens is characterized by significantly decreased egg production, liver steatosis and rupture, and sudden death [1]. As a kind of lipid metabolism disorder, FLHS is the main factor causing noninfectious mortality in caged layers globally [2,3]. Because of the expansion of the production scale and the intensification of the poultry industry, numerous laying hens suffer from FLHS, from approximately 5% during the regular production up to 20% in severe cases, which has a huge economic impact on the poultry industry [4]. The liver in birds, different from mammals, is the main organ for lipid metabolism; approximately 70% fatty acids (lipogenesis) are synthesized in the liver [5]. In addition, glucose transporter protein 4 (GLUT4), an important insulin-responsive transporter in mammals, is deficient in chickens, which may be associated with hyperglycemia and insulin resistance (IR) observed in chickens [6,7]. Furthermore, glucose concentration in birds is two to four times higher than that in mammals with the similar body weight [8]. The natural “insulin intolerance” makes birds more prone to both hyperlipidemia and hyperglycemia, which significantly increase the risk of liver damage [9].

There are multiple factors affecting the occurrence of FLHS in laying hens, including nutritional status, hormone homeostasis, toxic and harmful substances, and intestinal microbial composition (the enteric-liver axis) [10]. Overdosing on energy (a condition of nutrient excess) is the major factor causing the disease, and changing diets can efficiently prevent chicken FLHS [11,12,13]. Dietary supplements have been used for improving hepatic and blood indexes related to FLHS, which provides a new strategy for preventing FLHS occurrence rate in laying flocks [14,15,16,17,18].

Mammalian nonalcoholic fatty liver disease (NAFLD), also known as metabolic dysfunction-associated fatty liver disease (MAFLD), is a complex disease with many interacting metabolic pathways, especially the ones involved in lipid metabolism [19]. Similar to NAFLD, excess hepatic lipid deposition is a common pathological characteristic in birds with FLHS [20], and the pathogenesis of FLHS in laying hens is similar to that of mammalian NAFLD as well [21]. Therefore, laying hen has been recognized as an appealing animal model for investigating NAFLD in humans [22].

Despite the recently advanced studies in the understanding of the mechanisms of these two diseases (NAFLD in humans and FLHS in laying hens), the knowledge on the pathogenesis of the metabolic disorders is still incomplete. The “two-hit” hypothesis is inadequate to explain the disease-associated molecular and metabolic changes, while a multiple-hit hypothesis has been proposed [11], and IR is the theological center for the pathophysiological process of both NAFLD and FLHS [12], increasing hepatic fat accumulation by deposition of free fatty acids (FFAs), leading to over production of reactive oxygen species (ROS), and resulting in protein misfolding, autophagy inhibition, and mitochondrial damage within the hepatocytes [23]. These functional disorders challenge the hepatocytes with both oxidative and endoplasmic reticulum (ER) stress, mediating reactive oxygen/nitrogen species (ROS/RNS) [23]. Those factors likely act simultaneously to activate genetic and epigenetic mechanisms that contribute to the pathogenesis of NAFLD and its progression [11]. Although the exact pathogenesis of FLHS has not been fully elucidated, recent studies have revealed the similar pathological changes in the FLHS hens and found that FLHS can be prevented or determined by osteocalcin (OCN) [24,25].

## 2. Osteocalcin

### 2.1. Osteocalcin Gene and Protein

Osteocalcin, a vitamin K-dependent, osteoblast-derived noncollagenous protein, is commonly used as a marker of bone remodeling [26]. In 1995, chicken OCN RNA was sequenced from both the embryonic and adult chicken tissues, such as bone, brain, intestine, and kidney and probed with chicken OCN cDNA [27]. It has been confirmed that bone was the major site of OCN expression in vivo [27].

Chicken OCN protein contains 49 amino acids [27]. OCN presents in two forms: carboxylated (cOCN) and undercarboxylated (ucOCN). In chickens, cOCN is separated from ucOCN by post-translational modification, producing three γ-carboxyglutamic acids (γGlu) residing at positions 17, 21, and 24, respectively [27]. cOCN is deposited in bone to regulate bone development, while ucOCN has a lower affinity for hydroxyapatite (the mineral component of bone extracellular matrix), releasing into the circulation as a hormone to improve glucose metabolism [28,29]. The biological functions of ucOCN as the active form of OCN have been revealed in both human NAFLD and chicken FLHS [25,30].

In hens, the concentration of serum OCN significantly decreases with age [31,32], from 200–300 ng/mL at 6-week-old to 150 ng/mL at 16-week-old, then below 50 ng/mL after hens starting to lay eggs [24,33,34]. There is a difference in circulating total OCN concentration between adult hens and mice (about 300 ng/mL in mice), which may reveal some species-specific functions of OCN during sexual maturity in chickens, but this field needs more research [35].

### 2.2. Osteocalcin Receptor

The putative receptor of OCN, G protein-coupled receptor family C group 6 subtype A (GPRC6A), has been cloned from humans, mice, rats, and chickens [36]. Between humans and mice, the receptor sequence identity is approximately 80%, while it is higher (84%) between humans and chickens [37]. In humans, however, GPRC6A expresses in many organs such as the brain, lung, and liver. GPRC6A in chickens mostly expresses in the jejunum and liver [28,38]. The localization of ucOCN and GPRC6A on cell membrane has been demonstrated [39]. Both in vitro and in vivo genetic and pharmacological studies have confirmed that ucOCN acts as a hormone through activating GPRC6A [40]. Pi et al. [41] established the structural basis for ucOCN to activate GPRC6A. Teng et al. [42] provide the evidence of the interaction between ucOCN and GPRC6A and suggested that ucOCN tends to interact with GPRC6A via its N-terminus, while others hold the view that ucOCN interacts with GPRC6A via its C-terminus [41]. GPRC6A, however, has become the potential therapeutic target for regulating inflammation, metabolism, and endocrine functions [37,40,43]. Consistent with the previous studies, ucOCN protects high-fat-diet (HFD)-fed wild-type mice from obesity and NAFLD but does not have the similar function in GPRC6A LKO mice [36]. The current evidence demonstrates that GPRC6A directly mediates the influence of ucOCN in alleviating HFD-induced NAFLD in mice. In chickens fed with or without Selenium-enriched yeast (SeY), it has been identified that GPRC6A is the third Hub genes, suggesting that GPRC6A might play a vital role in liver metabolism and health in chickens [38].

In addition, there is, at least, another receptor of OCN, GPR158, which is mainly located in the brain and functions in cognition, stress-induced mood control, synaptic development, and hippocampal-dependent memory in mammals [44,45,46,47,48]. It has clarified that GPR158 gene is only present in vertebrates and highly conserved in chickens [47]. However, its functions have not been fully investigated in chickens.

### 2.3. The Function of Osteocalcin

**Figure 1:**
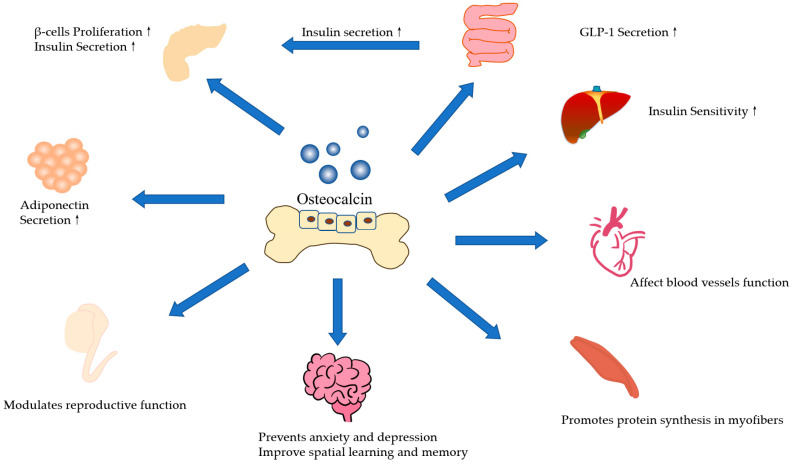
The diagram depicting the endocrine function of osteocalcin in different organs [59].

OCN has functions as a hormone in many organs, such as the brain [49], muscle [50,51], testis [52], liver [50], pancreas [29,53], gut [54], adipocytes [55], and blood vessels [26] in mammals (Figure 1). In chicken, circulating OCN has been clinically used as a specific biomarker of bone formation and osteoblast function [56]. Laying hens with keel bone damage [57] or fed a low phosphorus diet [58] have elevated serum OCN levels, which leads to an imbalance of bone homeostasis. On the contrary, the decreased its serum levels are related to bone health in older caged laying hens [33]. Generally, the functions of ucOCN in chickens are mainly concentrated on bone metabolism [57,58]. The recent results have showed that ucOCN may also affect egg production and eggshell quality in laying hens [31,34] and protect HFD-induced FLHS in aged laying hens through inhibiting excessive energy diet-induced metabolic disorder, oxidative stress, and related pathological damage by (1) accelerating the synthesis and secretion of insulin and alleviating insulin resistance; (2) decreasing accumulation of TG in hepatocytes; (3) decreasing inflammation and oxidative stress, and (4) enhancing autophagy [24,25].

Excessive deposition of triglycerides in the liver and inhibition of fatty acid oxidation result in the destroying of the homeostasis of lipid metabolism [60]. ucOCN functionally prevents accumulation of excess lipid in the liver, by which it reduces FLHS [60,61]. In addition, as the theological center of the process of FLHS, improving IR may reduce the incidence of FLHS [12,62]. IR leads to further hepatic damage triggered by oxidative stress and inflammatory reactions [12]. In addition, fat emulsion resulted in hepatic steatosis in chickens can be improved by administrating ucOCN via decreasing TG concentration and LDs number in hepatocytes [25]. Furthermore, ucOCN functionally alleviates mitochondrial damage, ROS synthesis, inflammatory cytokine formation, and hepatocyte apoptosis [25]. Meanwhile, ucOCN alleviates the decrease in autolysosome induced in high-fat-diet fed chickens [24]. The pathological changes are similar to the abnormality of autophagic function identified in NAFLD patients [53].

## 3. Molecular Mechanism of Osteocalcin in FLHS Chickens

The cellular mechanism of ucOCN regulating avian FLHS is still unclear, while it may be similar to the proposed signaling pathways identified in human NAFLD, including the mitogen-activated protein kinase (MAPK), nuclear factor-κB (NF-κB), hedgehog, AMP-activated protein kinase (AMPK), c-Jun-N-terminal kinase (JNK), and peroxisome proliferator-activated receptor (PPARs) [63,64,65]. Especially, recent studies show that ucOCN protects birds from FLHS via activating the JNK signaling pathway [25]. In addition, ucOCN may influence the developmental stages in FLHS chickens by activating GLP-1, AMPK, JNK, peroxisome proliferator-activated receptor α (PPARα), ADPN, Nuclear erythroid 2-related factor 2 (Nrf-2) signaling pathways [25,66,67,68,69].

### 3.1. Osteocalcin Reduces Fat Accumulation and Inflammatory Reaction by Inhibiting the ROS–JNK Signal Pathway 

**Figure 2:**
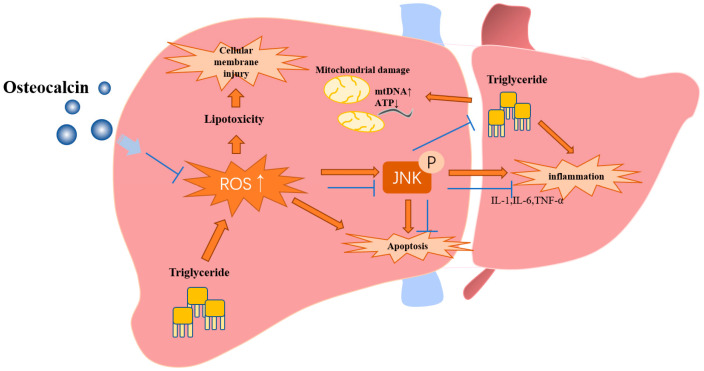
The diagram depicting the regulation of OCN effects on FLHS via the JNK signaling pathway [25]. ucOCN exerts its protective function through the ROS–JNK signaling pathway. In the liver, excess triglycerides lead to increased ROS synthesis, consequently increasing cellular membrane injury, hepatocyte apoptosis, and inflammatory reaction and upregulating the JNK signaling pathway. OCN functionally alleviates hepatocyte injury and associated mitochondrial damage, ROS synthesis, and inflammatory cytokine production by inhibiting the JNK signaling pathway.

Lipid accumulated excessively in the liver cells is a typical pathological character in laying hens with FLHS [12,70,71]. Excessive lipid storage in the hepatocytes (fatty liver) accelerates β-oxidation, increasing ROS concentrations (a class of undesired side products in cellular electron transfer reaction during nutrient metabolism) [72,73,74]. Numerus evidence have revealed that increased ROS activates many intracellular signal pathways including the JNK pathway [25,75]. The JNK is one of the members of the MAPK family [75], which is increased in both NAFLD patients [63] and animal models [76], abnormally mediating lipid overaccumulation, inflammatory response, and IR [75]. Blocking JNK may prevent the development of steatosis by the direct and indirect mechanisms [77]. For example, palmitic acid stimulates human hepatocytes to produce chemokine interleukin-8 (IL-8) via activation of JNK/AP-1 and NF-κB [78]. IL-8 is one of the key proinflammatory cytokines involved in modulating the inflammatory response, which initiates and/or enhances hepatic inflammation and injury [78]. In addition, the ROS–JNK signaling pathway is involved in IR, fat accumulation, apoptosis, oxidative stress, autophagy, and inflammatory reaction in mammals [49,79,80]. This reaction can be inhibited by administration of the ROS inhibitor (NAC) [25,81]. SP600125, the inhibitor of JNK, decreases pro-inflammatory cytokine interleukin-6 (IL-6) and TNFα and TG concentration, indicating that ROS–JNK induces inflammatory reaction and fat accumulation in chicken hepatocytes [25]. Similarly, the ROS–JNK signaling pathway can be suppressed by ucOCN in chicken hepatocytes [25]. It may suggest that the ROS–JNK signaling pathway is also involved in the functions of ucOCN in avian FLHS (Figure 2) [25].

### 3.2. Osteocalcin Might Prevent Insulin Resistance through the JNK Pathway

Recent studies have shown that IR is highly associated with FLHS [12,24,62]. In FLHS chickens, blood glucose tolerance, insulin sensitivity, and insulin concentration are decreased, and the relative mRNA expressions of genes of the insulin signaling pathway are disturbed [12,24]. ucOCN increases insulin concentrations, suggesting that ucOCN treatment may reduce HFD-caused IR in chickens [24].

Insulin, as a hormone, is responsible for glucose metabolism in the body [75,82]. In chicken liver, following bound to insulin, activated receptors stimulate insulin signaling (mainly the InsR/PI3K/AKT signaling pathway) to regulate energy metabolism [83]. IR occurs when cells are unable to respond to the transduction of signals derived from the interaction of insulin with its receptors [84], resulting from multiple factors, including increased FFAs [82]. Excessive FFAs produce harmful ROS with lipotoxicity in hepatocytes, leading to mitochondria function defection and decrease in insulin secretion [85]. FFAs also increase inflammation and impair insulin signaling through pro-inflammatory cytokines, such as tumor necrosis factor—alpha (TNFα) and IL-6, which are up-regulated in FLHS chickens [12,62,70]. IL-6 has been identified as a key DEGs in HELP-diet chickens, increasing JNK signaling pathway and suppressing cytokine signaling 3 (SOCS 3) [62]. Consequently, the activations of JNK and SOCS impede insulin-induced insulin receptor substrate (IRS) tyrosine phosphorylation, resulting in IR [86].

Animals lacking ucOCN have a low β-cell proliferation, glucose intolerance, and insulin immunoreactivity [87]. In both HFD diet-fed mice and (ob/ob) obese mice, inhibited JNK in the liver, muscle, and adipose tissue leads to enhanced insulin receptor signaling capacity and, consequently, enhanced insulin sensitivity [77,88,89,90]. In contrast, ucOCN decreases IL-6 and TNFα, which implies that ucOCN may alleviate IR through the JNK pathway in HFD chickens [24].

### 3.3. Effect of Adiponectin on Osteocalcin Protecting Poultry from FLHS

Adiponectin (ADPN) is one of the several hormones expressed in the adipose tissue, skeletal muscle, liver, diencephalon, testicle, and ovarian tissue in both mammals and chickens [91,92]. Its protein structure, gene expression, and function have been extensively studied and reported that ADPN has the potential to be used in treating a variety of obesity-associated diseases in mammals [93]. In NAFLD children, there is a positive correlation between ucOCN and ADPN [94]. In mice, ucOCN enhances ADPN release and improves glucose tolerance [95].

In chickens, similar to it in humans, upon binding to its receptors, ADPN activates the AMPK signaling pathway to enhance fatty acid oxidation and glucose utilization [93,96,97]. A negative association between ADPN and belly fat deposition in chickens has been reported [98]. Similarly, chicken recombinant ADPN ameliorates adipogenesis in oleic acid- and palmitic acid-treated LMH cells (Leghorn male hepatoma cell line) [99]. Pathologically, ADPN plays an important role in preventing the progression of simple hepatic steatosis to NASH as well as other liver diseases [100]. In NAFLD patients, ADPN is reduced in the blood and liver, while a high level of ADPN reverses stress-induced decrease in SOD activity with increased MDA and TG [101]. ADPN also inhibits inflammasome activation in hepatocytes [102] and alleviates the injury of NAFLD cells by reducing oxidative stress [103]. Activation of ADPN receptors has a protective effect on fatty liver injury in HFD-fed goslings through regulating the AMPK and p38 MAPK signaling pathways, as well as the transcription factors such as PPARα, reducing the lipid content in blood and liver tissues, improving antioxidant capacity, and regulating apoptosis and autophagy [104]. It further supports the hypothesis that AMPK ameliorates lipid metabolism through regulating the AMPK/SREBP1 pathway [105]. Sterol regulatory element binding protein 1 (SREBP1) and PPARα are two major transcription factors, playing a vital role in lipid synthesis and metabolism [105]. This result indicates that AMPK and PPARα have the similar physiological activities in regulating lipid metabolism.

**Figure 3:**
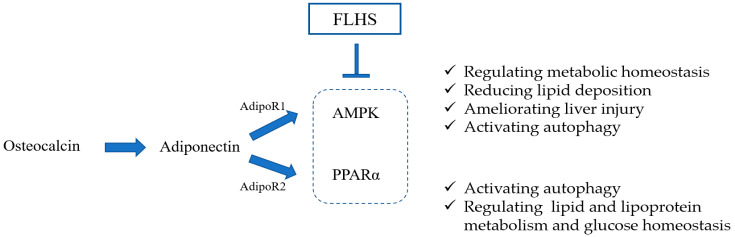
The diagram depicting the effects of osteocalcin on the pathways in the development of FLHS.

Taken together, the ADPN protects from NAFLD mainly through improving insulin sensitivity and reducing lipid accumulation and oxidative stress. Similarly, its functions may present in FLHS chickens. The facts that ucOCN regulating ADPN and ADPN affecting AMPK/PPARα raise the possibility that ucOCN activates ADPN in FLHS via the ADPN/AMPK and ADPN/PPARα signaling pathways, then affecting downstream metabolic pathways (Figure 3).

#### 3.3.1. Osteocalcin Protects Poultry from FLHS via the ADPN/AMPK Signaling Pathway

AMPK is an important energy sensor that regulates metabolic homeostasis [106]. The activity of AMPK is inhibited in FLHS since up-regulated AMPK is beneficial in preventing the occurrence of related metabolic diseases by affecting lipometabolism, autophagy, and oxidative stress [107,108].

AMPK exerts functions in lipometabolism. Atractylenolide III has been proven to reduce lipid deposition and ameliorate liver injury in HFD-induced NAFLD mouse model by activating the AdipoR1 down-streamed AMPK signaling pathway [69]. In poultry, AMPK signaling pathway regulates the lipometabolism in FLHS chickens, enhancing fatty acid catabolism and restraining fatty acid anabolism [109]. Similar results have been demonstrated in other studies [107]. The AMPK signaling pathway, for example, is involved in the process of hepatic steatosis in laying hens [110]. Activation of AMPK reduces lipid synthesis-related genes and protein levels, thereby decreasing the lipid production in hepatocytes [107,110]. Fu et al. [111] reported that maternally taking linoleic acid (CLA) supplementation mediates embryonic hepatic lipometabolism via the AMPK pathway.

Multiple evidence supports that autophagy plays an essential role in FLHS [24,112]. Autophagy maintains homeostasis by repairing or degrading damaged organelles and proteins [113]. Autophagy-related genes Beclin-1, Atg5, and Atg7 are suppressed in FLHS chickens [113], and the numbers of autophagosomes and autophagolysosomes decreased in liver of FLHS laying hens [30]. Aurantio-obtusin (AO) has been demonstrated to ameliorate hepatic steatosis via AMPK/autophagy and AMPK/TFEB-mediated suppression of lipid accumulation [114]. Up-regulation of the AMPK/mTOR signaling pathway activates autophagy to mitigate hepatic steatosis via promoting fatty acid oxidation [115,116]. mTOR, an important signal molecule downstream of AMPK, plays a central role in autophagy [117]. ucOCN may increase autophagy by regulating the ADPN/AMPK/mTOR signaling pathway in chicken FLHS.

**Figure 4:**
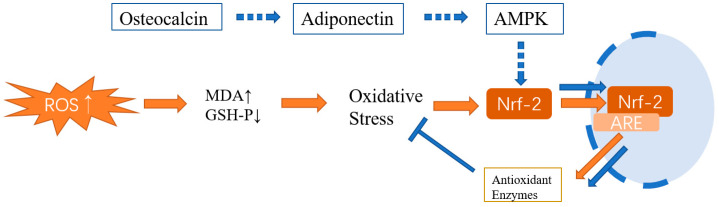
The diagram depicting the osteocalcin effect on regulating the Nrf-2 signaling pathway in the liver with FLHS.

AMPK may affect oxidative stress by activating the Nrf-2 signaling pathway. In the progression of FLHS, an imbalance between increased ROS synthesis and reduced capacity of antioxidant system induces oxidative stress [118]. Nrf-2 is considered a regulator of various antioxidant genes and detoxification enzymes against oxidative and electrophilic stress [119]. At homeostatic conditions, Nrf-2 binds to Kelch-like ECH-associated protein (Keap1) to form a Nrf-2–Keap complex in the cytosol [119]. When exposed to oxidative or electrophilic stress, Nrf-2 is translocated from the cytosol to the nucleus. In the nucleus, Nrf-2 binds to antioxidant-responsive elements (AREs) to promote numerous genes encoding a broad range of antioxidant enzymes [73]. Upregulation of nuclear Nrf-2 may contribute to the protective effect on the liver in developing FLHS [120]. Oleic acid (OA)-stimulation downregulates the Nrf-2 mRNA and nuclear protein levels in the primary chicken hepatocytes and LMH cells [121]. In addition, palmitic acid plus oleic acid (PO) treatment leads to markedly reduced nuclear Nrf-2 protein level, thus reducing the antioxidant capacity, which finally leads to a vicious circle of oxidative stress in the primary chicken hepatocytes [107]. Dysregulation of Nrf-2 may contribute to the developing of FLHS [121].Daily injection of ucOCN increases the nuclear Nrf-2 level in mice [73]. Pharmacological activation of Nrf-2 in HFD mice decreases the levels of IR, weight gain, TG, and ALT via regulating the related genes to reduce stress responses, such as ER stress and oxidative stress [122]. In HFD-induced FLHS hens, ucOCN decreases malondialdehyde (MDA, a toxic molecule) and increases glutathione peroxidase (GSH-Px, an important peroxidase) levels, indicating that ucOCN inhibits hepatic oxidative stress [24]. In addition, ucOCN may affect oxidative stress via the AMPK/ Nrf-2 signaling pathway. In primary chicken hepatocytes, AMPK prevents lipid metabolism disorders, oxidative stress, and inflammatory response through activation of Nrf-2 signaling pathway [107]. ucOCN may affect oxidative stress by regulating the ADPN/AMPK/Nrf-2 signaling pathway in chicken FLHS (Figure 4).

#### 3.3.2. Osteocalcin Protects Poultry from FLHS via the ADPN/ PPARα Signaling Pathway

Peroxisome proliferator-activated receptor α is a regulator of lipid and lipoprotein metabolism and glucose homeostasis [115]. It regulates mitochondrial β-oxidation and microsomal ω-oxidation involved in lipoprotein metabolism and inhibits lipogenesis through liver X receptor alpha (LXRα) and SREBP1 [16]. Similar to its function in mammals [115], PPARα exerts function in regulating the related pathways with lipid-lowering effect in chickens [16,123]. The activation of PPARα is suppressed in FLHS chickens [124,125]. Chen et al. [124] cloned and synthesized PPARα protein for producing chicken polyclonal antibodies and showed that the expression of PPARα was decreased in FLHS chickens. Zhu et al. [125] reported that high energy and low protein diet (HELP), one of the natural causes of chicken FLHS, dysregulated the PPARα signaling pathway, causing excessive accumulation of fat in the liver tissue with the potential to develop FLHS in chickens.

Activating PPARα may provide a new strategy to curb the development of FLHS in chickens. ucOCN influences the expression of PPARα by activating ADPN [94]. GW6471, a PPARα inhibitor, decreases AO-induced upregulation of PPARα in the hepatocytes [114]. Maternal CLA supplementation reduces fat deposition in chicken embryos, accompanied by an increase in PPARα protein concentrations and mRNA expressions in chicken embryos [111]. Furthermore, the PPARα signaling pathway affects autophagy. It has been reported that fenofibrate, a PPARα agonist, activates autophagy and reduces hepatic fat accumulation by upregulating the TFEB/TFE3 (the main regulators of lysosomal biogenesis and autophagy) [126]. In addition, Zinc (Zn) induces lipophagy in hepatocytes via activating the PPARα pathways [127].

### 3.4. Leptin

The sequences of chicken leptin mRNA and the full-length leptin receptor (CLEPR) cDNA have been reported [128,129,130]. Leptin expresses in chicken pituitarium, liver, brain, and duodenum, but not in the adipose tissue [131]. In chicken, leptin is operating at low level in an autocrine/paracrine fashion, which is usually undetectable in blood circulation [132].

Leptin down-regulates SREBP-1 and regulates the genes related to glucose metabolism, fatty acid, and lipid production [133]. By promoting hepatic triglyceride export and decreasing de novo lipogenesis, brain leptin protects from steatosis [134]. This function requires hepatic vagal innervation, suggesting that leptin exerts its functions in the liver at a central level.

Recently, Leptin has also been revealed to be involved in liver lipogenesis [135]. There is a positive correlation between ucOCN and ADPN in liver pathological changes in NAFLD children, while leptin has a negative correlation with serum OCN [94]. Modulating the leptin–adiponectin axis in hepatocytes and adipocytes using low molecular weight fucoidan and high-stability fucoxanthin (LMF-HSFx) has become a therapeutic approach to ameliorate hepatic steatosis, inflammation, fibrosis, and IR [136]. These findings further suggest that there is a potential correlation among ucOCN, ADPN, and leptin in NAFLD. However, whether the same correlation exists in FLHS chickens is unknown. In the future, more studies are needed to prove the direct effect of ucOCN on avian leptin.

Currently, the studies on avian leptin mainly focus on the reproductive system in growth and development [137,138,139]. In poultry, leptin activates the Janus kinase/signal transducer and activator of transcription (JAK–STAT) signaling pathway and regulates energy homeostasis through bending to the leptin receptor [140]. In addition, leptin affects autophagy in the hypothalamus, liver, and muscle in chickens by activating leptin receptor and the STAT pathway via AMPK mediation [141]. However, the effect of leptin on autophagy seems to be feed-independent, and the downstream signaling of AMPK remains to be explored [141]. In the future, more studies are needed to prove the direct effect of leptin on avian FLHS.

### 3.5. GLP-1

Glucagon-like peptide-1 in chickens is mainly released from the intestinal epithelial endocrine L cells (including the jejunum and ileum) stimulated by processing of proglucagon, protein, and amino acid [142]. The GLP-1 receptor (GLP-1R) also expresses in the brain and liver in chickens [143]. The receptor of putative chicken GLP-1 contains 459 amino acids in length and has high amino acid sequence which is identified with GLP-1R from humans, rats, and mice [143].

Functionally, GLP-1 inhibits food intake in both chickens and mammals [144,145]. Furthermore, GLP-1 has various extrapancreatic effects such as attenuation of hepatic lipid accumulation to prevent cardiovascular disease and ameliorate obesity [146]. GLP-1 and its analog liraglutide decrease lipid accumulation in primary cultured hepatocytes by increasing the phosphorylation of AMPK [147]. GLP-1 also directly regulates lipid metabolism in broiler chickens [147]. Furthermore, intraventricular injection of GLP-1 could reduce the level of blood sugar, affecting energy consumption and fat metabolism in chickens [148]. GLP-1 plays an important role in preventing avian from FLHS by regulating lipometabolism.

In mammals, ucOCN also promotes insulin release mainly through two methods: one is directly affecting pancreatic β-cells, and another is indirectly simulating GLP-1 secretion from the small intestine and then GLP-1 activating pancreatic β-cells to secrete insulin [54]. However, unlike in mammals, the receptor of GLP-1 has been identified in chicken pancreatic D-cells secreting somatostatin rather than in pancreatic β-cells [142,144]. Overall, whether ucOCN affects GLP-1 in chickens is unclear.

## 4. Conclusions

**Figure 5:**
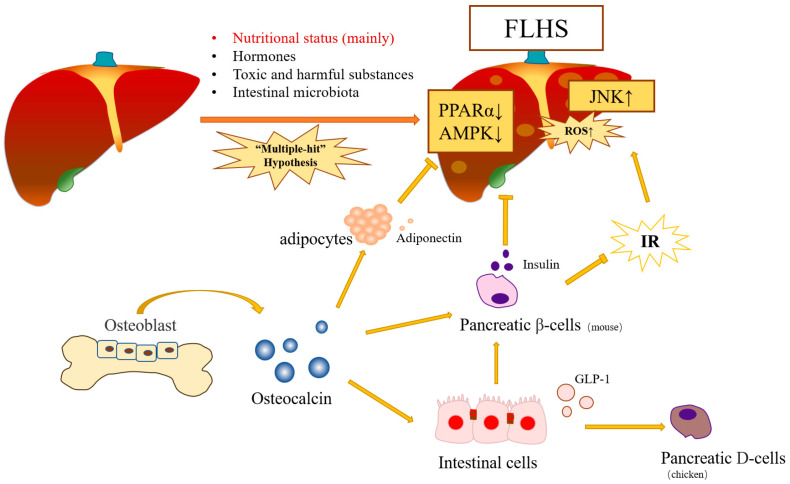
Diagram depicting the pathways involved in osteocalcin affecting FLHS in chickens. Osteocalcin derived from osteoblasts protects chicken from FLHS via (1) activating the JNK and AMPK signaling pathways and PPARα signaling down-regulation to reduce the effects of damaging factors (nutritional status, hormones, toxic and harmful substances, and intestinal microbiota) in the FLHS liver; and (2) activating pancreatic β-cells to stimulate insulin synthesis, thereby reducing insulin resistance and associated liver damage.

Multiple factors can lead to chicken FLHS, including nutritional status. The pathogenesis of FLHS in chickens has been proposed to be similar to the “multiple-hit hypothesis” proposed for mammalian NAFLD. As a multifunctional hormone, ucOCN protects laying hens from FLHS in various ways. Among them, the JNK signaling pathway is involved in inflammatory reaction and oxidative stress, and AMPK and PPARα prevent or reduce FLHS in chickens. While GLP-1 may indirectly affect FLHS via regulating insulin synthesis in pancreatic D-cells. The functions of ucOCN provide a therapeutic strategy for preventing FLHS in poultry (Figure 5).

## Data Availability

No new data were created or analyzed in this study. Data sharing is not applicable to this article.
